# Discharge to Assess: an evaluation of three case studies in the southeast of England to inform service improvement

**DOI:** 10.1136/bmjoq-2023-002515

**Published:** 2023-12-19

**Authors:** Stuart Jeffery, Jenny Monkhouse, Lavinia Bertini, Susie Walker, Rebecca Sharp

**Affiliations:** 1 Centre for Health Services Studies, University of Kent, Canterbury, UK; 2 NIHR Applied Research Collaboration Kent Surrey Sussex, Hove, UK; 3 Brighton and Sussex Medical School, Brighton, UK; 4 Kent Surrey Sussex Academic Health Science Network, Crawley, UK

**Keywords:** Community Health Services, Continuity of Patient Care, Organizational theory, Patient Discharge, Quality improvement

## Abstract

**Background:**

Discharge to Assess (D2A) emerged as a critical process during the COVID-19 pandemic facilitating patient flow within hospitals, however research on the post-discharge community services of this pathway remains limited. We conducted an evaluation to examine the impacts, capacity, processes and barriers associated with D2A and to identify best practice across three sites in the southeast of England.

**Methods:**

We interviewed 29 commissioners, providers and staff members involved in the delivery of D2A pathways within three Health and Care Partnerships. Framework analysis of the collected data revealed three prominent themes: the commissioning of services encompassing funding, structure, culture, and expected outcomes; multidisciplinary collaboration including staff skills, team connections, and coordination; and information and knowledge exchange such as assessment methods, record management, and availability of operational insights.

**Results:**

62 specific enablers and blockers to effective D2A practice emerged.

**Discussion:**

These findings supported the development of a comprehensive service improvement toolkit.

**Conclusion:**

Five recommendations are proposed: 1. Examination of pathways against the 62 enablers and blockers to identify and resolve pathway obstacles; 2. Establish a local operational policy accessible to all providers; 3. Enhance coordination and communication among service providers, patients and carers; 4. Strengthen oversight of service user flow; 5. Develop a consistent Patient Reported Outcomes Measure to facilitate feedback and service enhancements for individuals discharged from urgent care pathways.

**WHAT IS ALREADY KNOWN ON THIS TOPIC**

- Discharge to Assess was given significant prominence by the National Health Service at the start of COVID-19 and continues to play a significant role in enabling the flow of patients through hospitals.
- There is a limited amount of evaluative research on Discharge to Assess and very little that considers the community part of the pathway.

**WHAT THIS STUDY ADDS**

- This evaluation sheds light on the experiences of both health and social care commissioners, providers and staff involved across the D2A pathway.
- This evaluation emphasises the importance of consistent policy implementation, improved communication, enhanced oversight and the integration of outcome measures to drive service improvement.

**HOW THIS STUDY MIGHT AFFECT RESEARCH, PRACTICE OR POLICY**

- 62 specific enablers and blockers were identified that may support service improvement of D2A pathways.

## Introduction

Directors in the Kent, Surrey and Sussex Integrated Care Systems (ICSs) had identified the Discharge to Assess (D2A) pathway as a key service change and priority to enable and increase the flow of patients through hospitals and aid post pandemic recovery. They felt that the pathway had been implemented differently in different places and that there was a need to ensure that the learning from these implementations was identified, shared and used for service improvement.

Improving hospital capacity is essential, especially during a major incident such as COVID-19.^1^ In March 2020, at the start of wave 1 of COVID-19, the Department of Health and Social Care introduced the first national policy and associated funding for D2A.^2^ While D2A had been implemented locally in some areas prior to this, this heralded the first national framework for D2A.

The D2A pathway aims to reduce the length of hospital stays for patients who are clinically optimised but require a degree of care and/or support to fully recover. There are two key differences between a D2A pathway and traditional discharge pathways. In D2A:

1. Patients are discharged either back to their homes or into a community setting (eg, a care home) where they receive up to 4 weeks of National Health Service (NHS) funded care, reablement and rehabilitation (not traditionally funded by the NHS).
2. The long-term assessment of needs is made in non-hospital settings once recovery is maximised, rather than prior to discharge from the acute hospital.

**Figure 1:**
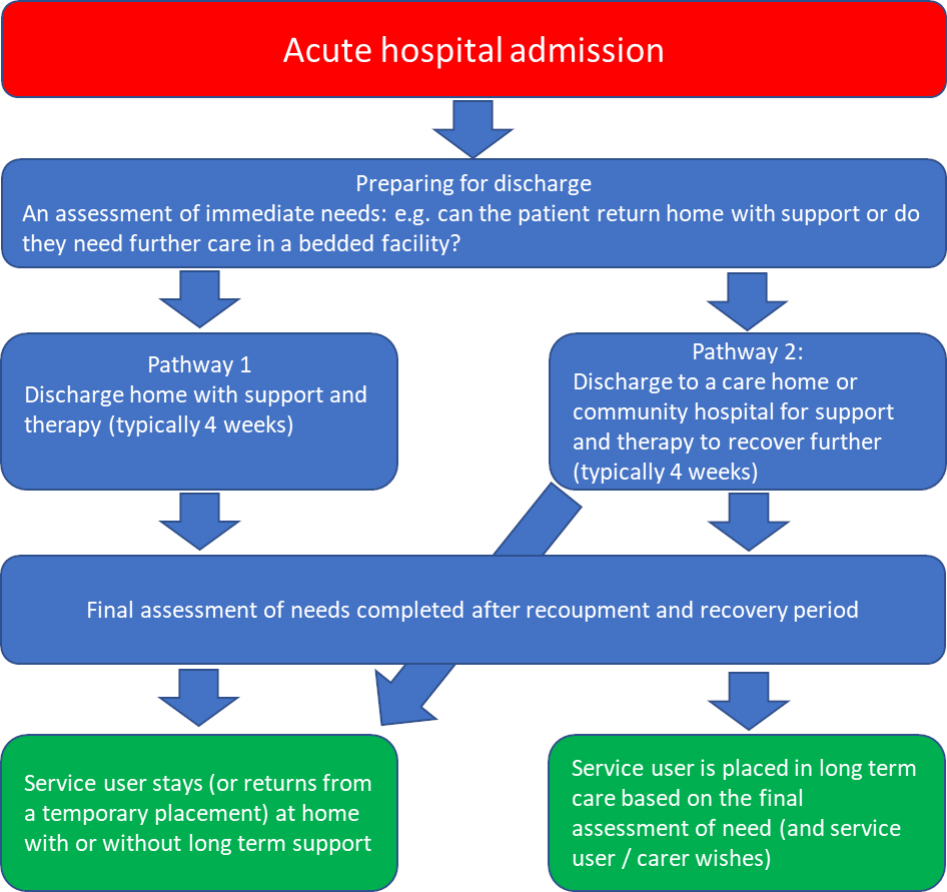
Typical Discharge to Assess pathway

D2A uses pathways 1 and 2 of the four pathway model of hospital discharge,^2^ shown in figure 1. Pathway 1 is discharge home with additional support and pathway 2 is discharge to a temporary bedded facility for further rehabilitation, recouperation and reablement. Pathway 2 leads to either a return home or to a longer term care home placement. Pathway 0 is for simple discharges for those requiring no changes to care and pathway 3 is for those requiring long-term complex or end of life care; neither of these feature on the D2A pathway.

While learning from the recent expansion of D2A is not well documented, the ideas underpinning D2A have existed for some time, for example, the benefits of early supported discharge for patients who had a stroke are well documented^3 4^ and evidence from earlier versions of D2A suggestion benefits such as an average reduction in length of stay.^5^ In addition, Gadsby *et al*^6^ found issues around the selection of patients for the pathway by hospital staff, power imbalances that hampered service improvements, and a lack of delivery of anticipated benefits. The motivation to reduce length of stay was felt to override the need for person centred care.

Studies based on evidence after the introduction of the national policy^2^ are rarer. An evaluation by RSM Consulting^7^ reviewed 10 systems across England and found significant variation in approach; however, there was agreement on the need for single points of access, a shift of resources into the community, integrated teams and additional community capacity. Their evaluation focused more on the initial part of the pathway than the community aspects and they highlighted problems with data management (ie, knowing what was happening/oversight), uncertainty of funding, and communication with families. They suggested that the funding of care for the initial post discharge period was helpful, partnership working enabled good relationships, adaptability of workforce was present and that systems were less risk averse than before. In addition, the pilot study to this project,^8^ which focused on the experiences of hospital staff, showed benefits to the quadruple aims of healthcare improvement (improved quality, efficiency, wider health benefit and staff satisfaction^9^), particularly around staff satisfaction and working conditions with staff reporting enhanced autonomy and widening skill sets.

From a broader perspective, a few earlier studies on hospital discharge have focused on the coordination of professional groups in discharge planning^10–14^ and on professionals’ perspectives.^15–17^ These studies concur that effective communication between hospital and community care settings is an important element for quality discharge.^18^ Also identified are barriers and enablers to coordination and communication across groups and settings. Waring *et al*^11^ looked at the organisational and professional boundaries that ‘define and separate professional groups’ (p.41) to understand their effect on the safety and quality of discharge. They concluded that professional boundaries are a threat to safe discharge and that promoting a culture of collaboration improved the quality of discharge and resulted in further satisfaction for the different occupational groups involved.

It is considered that hospital discharge should not be a single event but rather a complex process of care transition that require effective communication and coordination between heterogenous groups^10 19^ across health and care settings, such as hospital teams, general practitioners, community nurses, social care workers, patients and families.^20^ Integrated care, joined-up health and care services to deliver person-centred care, is understood to help facilitate the transitions across organisational boundaries^21^ and the wide variety of players and complexity that can exist in a person’s transfer of care from an acute setting to a long-term place, either at home or in a care home, requires coordination and integration. This coordination can be facilitated through information flows that support decision making and through colocation.^22^ Communication is not just important between teams but poor communication with service users and carer reduces the effectiveness of care transitions.^23^ Shared multidisciplinary documentation improves interprofessional, integrated and multidisciplinary working^24–26^; however, funding and interoperability difficulties can prove to be barriers.

While much has been written on the integration and communication in discharge pathways and there has only been one significant study on D2A which focused predominantly on the predischarge elements; evidence on the postdischarge community-based services of the D2A pathway specifically is scarce. After discussions with NHS England, we agreed to focus on this element.

## Methodology

### Context and aims

An overarching D2A project was set up by the NIHR Applied Research Collaboration Kent Surrey Sussex and the Kent Surrey Sussex Academic Health Science Network with three aims: (1) evaluating barriers and enablers across the pathways, with a focus on post-discharge community-based services including NHS community services, social care services and Voluntary, Community & Social Enterprise sector; (2) understanding the experiences and outcomes of service users and informal carers; (3) developing outcome and process measures.

In this paper, we present findings from semistructured interviews with staff, providers and commissioners in hospital and community-based services related to the first aim.

## Design

We used qualitative, semistructured interviews to gain insights on commissioners’, providers’ and staff’s experiences and views on the implementation of the D2A pathway. Qualitative interviews were chosen as they allow to explore in-depth a range of experiences and perceptions of the different actors involved across the pathway.^27^

A topic guide was codesigned with Patient and Public Involvement advisors and stakeholders (eg, hospital and community-based services staff and providers) from the three case sites in a series of three workshops. The topic guide was designed to explore interviewees’ experiences and views on the processes, barriers and enablers to delivering D2A and the effectiveness and sustainability of the pathway. The design allowed to explore and evaluate three different levels: (1) general views on D2A as a pathway of hospital discharge, (2) experiences and views related to implanting D2A locally and (3) examine topics pertinent to the interviewee’s role within the pathway.

The topic guide focused on postdischarge services and transition of care from hospital to community-based services. Questions related to acute care were also included for context and to understand this important interface for care transition. Recommendations for improvement were also discussed.

### Sites and participants

The three case sites studied within the overarching evaluation were selected with the following criteria in mind.

- One each from Kent, Surrey and Sussex.
- Willingness and capacity to participate.
- Availability of key metric data, such as ‘Delayed transfers of care’, ‘unplaced hours’, and so on.
- A mix of rural and urban setting.
- Different social care commissioners/providers.
- Varying levels of deprivation.
- Preference of ICSs.

Participants included staff from acute trusts, community healthcare services, social workers, home care providers, care home providers and members of the voluntary/third sector, plus health and social care commissioners. We aimed for one person from each type of provider and commissioner in each site. The people interviewed were predominantly from the community rather than hospital; however, hospital staff were included to ensure the complete pathway was covered.

### Data collection

Semistructured interviews were conducted by two researchers, JM and LB, with commissioners, providers and staff, from both hospital and community settings, involved across D2A pathways 1 and 2. Interviews were conducted between March and June 2022 on MS Teams, lasted between 45 and 90 min, and were audio recorded.

Local operational policies were requested from leads in each case site.

### Data analysis

Interviews were transcribed and anonymised. Data were analysed by two researchers, JM and LB, using framework analysis^28^ which consists of five stages: familiarisation, identification of a thematic framework based on the interview topic guide, indexing, charting and mapping, and interpretation across the research team. Identification of themes was both inductive and deductive. The data from each case site were first coded separately by JM and LB to identify themes and processes specific to each site. This was followed by a comparison and discussion of themes across the three case sites to identify overarching themes. At this stage, codes and themes were discussed and agreed on by the whole team. The charting and mapping were conducted using the qualitative data analysis software QSR NVivox64.

### Patient and public involvement

Patient and public involvement was central to the wider evaluation from the start. We recruited a Patient Advisory Panel to guide and advise researchers. Meetings occurred each quarter which included the entire project team and were reported to the project board. The panel provided feedback and supported the co-production of study material (eg, participant information sheet, interview topic guides) and outputs, to ensure the service user and carer voice was heard and understood.

### Findings

A total of 29 members of staff from across each of the three case sites were interviewed using the semi-structured set of questions.

Three core themes were identified by the research team in the cross-case analysis. Each core theme had three subthemes and each subtheme had a range of issues that related to enablers or barriers to the effective running of a D2A service.

A detailed list of 62 enablers and barriers were identified within the themes. Expressed as questions, these supported the development of a service improvement toolkit (see table 1).

**Table 1:** Enabler and barrier questions

Theme	Subtheme	Question
**Commissioning**	**Finance**	Is the funding sufficient to provide capacity to meet the demand?
		Is there capacity to provide care after the D2A period?
		Has capacity to bridge care been commissioned?
		Is there agreement for longevity to ensure that the service is stable?
		Have out of area agreements been made?
		Is there support for recruitment?
		Has weekend support been commissioned?
		How are providers paid, particularly care homes as there are complaints and delays?
		Is there easy access to equipment?
	**Structure and culture**	Is there a clear strategy for the service?
		Has the team been built with a clear culture?
		Does the team understand the purpose and principles of D2A?
		Has there been any training on D2A?
		Does the team operate as a single (or virtual single) team across the length and breadth of the pathway?
		Is the home first principle being met?
		Have barriers between teams been removed ensuring that the team works as a whole rather than passing patients and requests between silos?
		Is the service patient centred?
		Is there access to equipment and home changing / furniture moving?
	**Outcomes**	Have outcome requirements for the service and their monitoring been built in?
		Is there a process for accountability and assurance?
		Are outcomes for the service defined? Are they monitored? Are they reported?
		Does service user and carer experience shape pathway development?
		Is there transparency of outcomes, process and need across the system?
**Multidisciplinary working**	**Connections**	Are the different players in the pathway connected?
		Do health and social care work together or are there boundaries?
		How does one part of the pathway know what others are doing?
		Have silos been broken down and does the team work as a virtual team?
		Do community services have a strong voice?
		Is there a culture of development and integration?
		Is the service flexible and agile?
	**Skills, knowledge and understanding**	Does the team include a range of therapists and other skills?
		Has the team been trained in therapy and rehab skills?
		Does the team know what other disciplines do?
		Does the team have access to resolve housing problems (eg, homelessness and hoarding)?
		Is specialist mental health support available?
		Have there been assessments of the risks in care homes and at home for service users with challenging behaviour?
		Capacity for dealing with people with complex needs?
		Are the needs of people with dementia understood?
		How are carers’ needs addressed?
	**Coordination**	Are there single points of contacts for key workers, coordinators, service users and carers?
		Are there huddles and MDT meetings?
		Is there a hub for the coordination of the service and care?
		How are different perspectives on care and need managed?
		Is there continuity of care as patient moves through pathway?
		How are risks and safeguarding coordinated and managed?
		How is the third sector capacity and involvement managed.
		Is there a directory of resources?
**Information**	**Assessments**	Do assessments start with essentials for discharge and increase in detail during the pathway?
		Is the assessment tool agreed by all parties? Do people have the skills to complete it?
		Does the information flow through the pathway? How is it shared?
		How are service users, carers and family expectations discussed? What information are they given? Is there an agreed set of information / leaflets?
		Do discussions with service users, carers and staff bring forward creative solutions? Is there an understanding of the benefit of not being in hospital?
		How is risk assessed? Are risks understood by both acute and community staff? What level of experience and skill sharing is in place?
		How is the initial level of care needed identified and agreed? Is that level of care able to be changed quickly after discharge? How is this communicated with the service user and carer?
		Are service users aware of what will happen at the end of the D2A period?
	**Information management**	Is there a single dynamic patient record? Is there a single assessment and recording process?
		Are records electronic and shared?
		Do all staff involved in the pathway have access to the electronic record? Can they both read the information and write to the record?
	**Oversight**	Do key workers and managers know who is doing what and when?
		Are service managers, system managers and commissioners sighted on available capacity and the flow of service users through the pathway?
		Are service managers, system managers and commissioners able to monitor the pathway across system including waiting lists and capacity?
		Is information on outcomes used to drive improvements?

D2A, Discharge to Assess.

### Commissioning

#### Pathway funding

Ensuring that the right level of funding and resource was provided to the service to meet demand was an obvious need but there were other aspects to the financing and resourcing of the pathway (including staff and equipment) that were found. The D2A pathway does not exist in isolation and the care of a service user is often passed from one provider to another. It is therefore important that follow on services have sufficient capacity to accept referrals, which is sometimes complicated by the use of the same staff for both long term care and for the short-term D2A period.

“We end up with a lot of people who stay on the Discharge to Assess service much longer than … the agreed amount” (Participant 1-3)

Participants felt that the pathway should ideally operate 7 days a week, in line with the rest of urgent care.

Agreements and processes for invoicing were found too difficult for some providers, and for patients who live out of the area of the Local Authority, a need for a working arrangement with neighbouring LAs was expressed.

Longer contracts were felt to provide stability for both team members and providers.

“…it’s that long term look as opposed to … a panic about capacity because of winter pressures” (Participant 1-3)

#### Structure and culture

A clear structure and embedded culture were felt to be enablers to a good D2A pathway; however, there was a lack of operational policy or strategy for any of the three case sites reviewed. This, combined with low levels of training, was felt to be problematic. While the high-level principles of D2A were broadly understood, staff generally felt more training and a move towards working as a single or virtual team would help with removing barriers and enhancing discussions. Generally, people understood the home first principle which they tried to follow.

“it has been a cultural shift for many teams and that has been… challenging” (Participant 2-2)

Community and social care services wanted a stronger say in the development of the pathway, rather than being passive recipients. There was a feeling that a flexible and agile culture was helpful on this pathway.

It is worth noting that each site was asked for documents or a policy that described the local pathway but none were provided. Some participants suggested that they were not aware of such a document.

#### Outcomes

Outcome requirements for the service and related monitoring had not been considered by commissioners in significant depth and while some aspects of reporting were described, this was generally felt to be an area that could be improved. In particular, the use of service user and carer feedback was not widely used in service improvement work.

“in terms of outcomes for the client, I think that’s gotten lost in we just need to get them out of hospital” (Participant 3-7)

### Multidisciplinary working

#### Skills, knowledge and understanding of staff

It was felt to be helpful to have access to and include in the pathway, the skills, knowledge and understanding of a wide range of therapists, including access to mental health advice from dementia support teams. This was considered problematic in most teams particularly around the support for people with dementia. Also, unsurprisingly, service users with complex needs required a significant amount and range of resources, for example, dietetics, occupational therapy and specialist nursing.

“it’s very hard to do D2A when you don’t have everything you need in the community” (Participant 2-4)

It was felt risk assessments could be reviewed particularly for service users with challenging behaviours. Being able to manage complex housing needs was discussed by many, for example, homelessness and hoarding.

#### Connections between the teams

The need for robust connections between teams and stages in the pathway was highlighted, particularly as the pathway often crosses from acute care into community health, and then into social care. Removing these boundaries and ensuring that D2A staff members understand the functions and processes of adjoining services could reduce silo working and foster the culture of integration desired by some participants.

“We have a daily conference call … with health, it’s with therapy, … it’s with social services” (Participant 1-8)

Integration, that is, the creation of virtual teams spanning providers and sectors, was felt to be enhanced through colocation, culture development and a shared sense of purpose.

#### Pathway and team coordination

The coordination of care was felt to be essential. Generally, pathways had some form of hub or discharge team that facilitated the initial move from the acute hospital and most pathways had some form of huddle or MDT meeting to discuss and coordinate care. Single points of contact (SPOC) existed to some extent but there was a feeling that these could provide better communication for both team members and for service users/carers. A SPOC might be combined with a directory of resources given the variability and complexity of needs; this may also support access to third sector community support. Finally, there were many concerns about continuity of care as service users move through the pathway.

“the passing off of the patient between health and social care [is] where it falls apart” (Participant3-13)

### Information

#### Assessments

Assessments that start with the basic information required for discharge and increase in detail during the pathway were felt to be in line with D2A principles. Participants felt that this and the assessment documentation itself need to be agreed by all parties. Assessments were not always considered to be completed correctly, this was reported be due either to a lack of understanding of the key information needed or simply a lack of time.

“information from the hospital has been really poor… key bits of information were missing” (Participant 3-10)

Participants explained that it sometimes appeared as though service user, family or carer expectations had not been managed effectively. It was felt that open discussions with service users and carers can help bring forward creative solutions to help with resolving issues on an individual’s pathway.

Concerns about the assessment of carers needs were noted and there was wide recognition that the views and needs of carers needed more prominence in assessment.

Staff described a disparity in the understanding and implementation of risk assessment, management and communication across the D2A pathway. This sometimes manifested as the wrong levels of care being provided initially which can lead to a risk of injury for staff and service users or an unnecessary level of care.

#### Management of the records

Participants expressed the need for a single, dynamic service user record. It was felt that the introduction of such a record system, available and editable by all teams across the D2A health and social care boundaries, would facilitate smoother transitions in care.

“there’s twelve different referral routes, eight different information systems” (Participant 3-10)

### Operational oversight

There were also concerns that individual services and teams do not have oversight of the whole pathway. A need for key workers and managers to know who is doing what and when, and for service managers, system managers and commissioners to be aware of available capacity and the flow of service users through the pathway, became apparent. There was a desire to use this oversight alongside outcome information, to drive improvements.

“we have spreadsheets upon spreadsheets” (Participant 2-3)

“there’s a lot of work still to do… in terms of tracking, monitoring, reporting” (Participant 1-1)

## Discussion

This study explored the implementation of three D2A pathways emphasising their significance in facilitating the flow of patients through hospitals and supporting post-pandemic recovery efforts. It is evident from the literature that delayed hospital discharges pose considerable challenges within the NHS in England,^29^ leading to adverse health outcomes, increased mortality and financial costs.^30–32^ The introduction of a national policy for D2A^1^ in response to the COVID-19 pandemic marked a significant shift in the approach to hospital discharges.

The national policy introduced NHS short-term funding of increased care following discharge and long-term assessments being conducted once the person has maximised their recovery. This should result in shorter hospital stays and better long-term assessments conducted in more suitable environments. While evidence of shorter lengths of acute hospital stay were not explored in this study, there was little evidence found of better long-term assessments or outcomes at the end of the D2A period.

The post-2020 evidence regarding the national policy’s impact is limited, but it does suggest variations in approaches, emphasising the importance of single access points, integrated teams, and the need for increased community capacity,^7^ all of which were echoed by this study. Additionally, the study’s pilot project^8^ found benefits in terms of staff satisfaction and working conditions however these were not strong themes in this current study.

This study identified three key themes, the importance of information flows and communication, how multidisciplinary teams work within an integrated pathway, and the structure and culture of the pathway, that is, how it is commissioned plus the impact of the national policy on the D2A pathway.

These themes align with previous research on hospital discharge,^10 11 13 14^ particularly underlining the importance of effective communication and coordination among various healthcare professionals across different care settings. Professional boundaries and a lack of collaboration can hinder safe and quality discharges; thus, integrated care and seamless communication are critical in the complex process of care transition. However, although the national policy^2^ has played a helpful role in funding and standardising the D2A pathway, the absence of local operational policies hinders the integration process, creating ambiguity and challenges. There is a clear need for further embedding consistency and understanding within healthcare teams to navigate the pathway’s complexity, as suggested by The Kings Fund.^21^

While the theme of how services are commissioned captures the concepts of strategy, culture and integration, it also covers capacity of the service, that is, how many service users can be catered for and how they flow through the pathway. Capacity constraints are widespread in health and social care,^33^ requiring national-level solutions. Nonetheless, this study found that some managers and commissioners have reported difficulties in accessing information related to capacity and blockages, suggesting a need for improved oversight of service-user flow.

In summary, having a local operational policy, building a culture of integration and improving communication should help the effective implementation of D2A. Additionally, improving capacity and oversight of the flow of patient should help the management of the pathway.

### Recommendations

The five recommendations from this part of the evaluation are:

- That the service improvement tool developed from the findings is used to help identify and resolve blocks in the pathway.
- A local operational policy for the pathway should be available to all providers on the pathway.
- Coordination and communication across all service providers and with patients and their carers requires improvement.
- Oversight of the flow of service-users needs development: at a wider level than individual Health and Care Partnerships there is work to integrate live data on flow and capacity into existing oversight tools to ensure operational oversight is possible.
- Developing a consistent Patient Reported Outcomes Measure for people discharged from an urgent care pathway to aid feedback and service development.

### Limitations

It is important to acknowledge that the generalisability of the findings may be limited to the specific locations and healthcare settings examined in the study. The sample size of 29 staff members, while providing valuable insight, will not fully encompass the range of perspectives held by the larger population.

The absence of documented local pathway descriptions hinders a comprehensive understanding of the contextual factors and guidelines that inform the implementation of D2A pathways.

It is worth noting that this element of the evaluation primarily focuses on the qualitative analysis findings, while the comparative analysis of D2A documentation alongside the objective outcome measures is to be presented in a separate publication. Patient and carer experiences will also be reported on in a further article.

## Conclusions

We interviewed commissioners, providers and staff involved in D2A pathway to determine the findings presented in this paper have highlighted key areas that contribute to and inhibit an effective D2A pathway. While there is reasonable consistency of approach across the three case sites, there are also processes that are managed better in some sites than in others. It is likely that this variability across sites is present at a national level and that a service improvement tool (now developed and available at https://research.kent.ac.uk/evaluating-discharge-to-assess/) would benefit other sites too. Based on this evaluation, we also identified the four following recommendations for D2A to be effective in practice: ensuring a shared understanding of local processes, (ie, an operation policy), maintaining high standards of communication both between teams and with patients and carers, having an operational oversight of the pathway, and measuring outcomes for service users and carers to facilitate continuous improvement.

This study provides valuable insights into the implementation of D2A pathways laying the groundwork for further investigation and improvement in this area.

### Other information

Funding for the evaluation was provided by the NHS Insights Prioritisation Programme, as part of the Accelerated Access Collaboration.

## Data Availability

Data are available upon reasonable request.
